# Recent advances in understanding frontotemporal degeneration

**DOI:** 10.12688/f1000research.20330.1

**Published:** 2019-12-13

**Authors:** Barbara Borroni, Alberto Benussi

**Affiliations:** 1 Neurology Unit, Department of Clinical and Experimental Sciences, University of Brescia, Brescia, 25100, Italy

**Keywords:** frontotemporal dementia, biomarkers, Tau, TDP43, trials, treatment, diagnosis

## Abstract

Frontotemporal degeneration (FTD) is a heterogeneous spectrum of neurodegenerative disorders characterized by diverse clinical presentations, neuropathological characteristics, and underlying genetic causes. In the last few years, several advances in the knowledge of clinical and biological aspects have been accomplished and three major scenarios have emerged that will represent the core issues in the FTD scene over the next few years. Foremost, the development of cerebrospinal fluid and blood biomarkers as well as neuroimaging techniques will aid the pursuit of new diagnostic and prognostic markers able to identify the ongoing proteinopathy and predict disease progression, which is key in identifying and stratifying patients for enrolment in clinical trials as well as evaluating response to treatment. On the other hand, current research has focused on the first attempts to slow down or revert disease progression, with the identification of disease modulators associated with disease onset and the ongoing development of the first pharmacological treatments for both sporadic and genetic FTD. Future research will certainly improve our knowledge of FTD and possibly open up a new era of disease-modifying therapies for this still-orphan disorder.

## Introduction

Frontotemporal degeneration (FTD) is a highly heterogeneous spectrum of neurodegenerative disorders, not only clinically but also genetically and neuropathologically ^1^. This significant heterogeneity has important implications for both diagnostic and therapeutic purposes. Considered in the past as a rare disease, FTD has been clearly shown by recent epidemiological studies ^2^ and the refinement of new clinical criteria ^3, 4^ to be much more frequent than previously thought.

In the last few years, a giant step forward in the knowledge of clinical and biological aspects has been accomplished. Clinically, FTD is characterized by behavioral abnormalities, language impairment, and deficits of executive functions ^5, 6^. According to newly revised criteria, three different clinical variants have been defined: the behavioral variant of FTD (bvFTD) ^4^, the agrammatic variant of primary progressive aphasia (avPPA), and the semantic variant of PPA (svPPA) ^3^.

One of the most compelling chapters in the recent literature of FTD is represented by the identification of causative mutations responsible for monogenic autosomal dominant disease. Several genetic mutations have been associated with FTD, the most common being those in the microtubule-associated protein tau ( *MAPT*) gene, the granulin ( *GRN*) gene, or the expansion on chromosome 9 open reading frame 72 ( *C9orf72*) gene ^7, 8^. However, other genes are associated with rare FTD cases, such as valosin-containing protein ** ( *VCP*) mutations, which are linked to a specific condition called inclusion body myopathy with Paget disease of the bone and FTD (IBMPFD) ^9, 10^, charged multivesicular body protein 2B ( *CHMP2B*), which is involved in the endosomal–lysosomal pathway ^11, 12^, fused in sarcoma ( *FUS*) ^13, 14^, TAR DNA-binding protein ( *TARDBP*) ^15, 16^, sequestosome 1 ( *SQSTM1*) ^17– 20^, TANK­binding kinase 1 ( *TBK1*) ^21– 24^, and ubiquilin 2 ( *UBQLN2*) ^25– 27^.

Along with heterogeneity in both clinical presentations and different autosomal dominant inherited genetic traits, diversified neuropathological hallmarks may be responsible for distinct frontotemporal lobar degeneration (FTLD) diagnoses, which are constituted primarily by tau or TAR DNA-binding protein 43 (TDP-43) depositions ^28, 29^. Therefore, the critical issue in the current literature of FTD is the lack of a clear-cut relationship between clinical phenotype and neuropathological features. Up to now, the ongoing neuropathological process, and consequently the underlying pathogenetic mechanisms, may be predicted only in monogenic disease. In this case, it is well established that *GRN* mutations and *C9orf72* expansions are associated with FTLD-TDP neuropathology while *MAPT* mutations are associated with FTLD-Tau ^30^. Recent work has also explored the structural and functional neural correlates of behavioral symptoms in FTD. Among these studies, a few have focused on the gray matter correlates of apathy and disinhibition ^31– 34^, while others have attempted to connect the white matter associations with behavioral symptoms ^33, 35– 37^.

According to these premises, three major scenarios have come out of the recent advances in the field and will represent the core issues in the FTD scene over the next few years. On one hand, the search for diagnostic and prognostic markers able to identify the ongoing proteinopathy in FTD and predict disease progression will be key in identifying and stratifying patients for enrolment in clinical trials. On the other hand, current research has focused on the first attempts to slow down or revert disease progression, with the identification of disease modulators associated with disease onset and the ongoing development of the first pharmacological treatments in a currently considered orphan disorder.

## Looking for neuropathology and prognostic markers

In the past few years, many efforts have been devoted to finding reliable markers which reflect the ongoing neuropathological process in non-genetic FTD. These markers are critical for evaluating potential disease-modifying treatments targeting either tau or TDP-43 pathological mechanisms in homogeneous groups, independently of clinical phenotype, and to better understand the disease pathophysiology ^38^.

Hu and colleagues proposed the cerebrospinal fluid (CSF) phospho-tau _181_ to total tau (p/t-tau) ratio as a viable biomarker to identify FTLD with TDP-43 pathology as compared to FTLD-Tau, with a reduced ratio suggestive of TDP-43 pathology ^39^. These findings have been confirmed in a few subsequent studies in FTD ^40– 44^, as well as in amyotrophic lateral sclerosis (ALS), which frequently is associated with TDP-43 pathology ^45^. Conversely, a recent report investigated novel CSF tau fragments (N-123, N-mid-region, N-224, and X-368) in both FTLD-TDP and FTLD-Tau; however, none of the novel tau species showed a significant difference between pathological groups ^41^. Similarly, CSF and plasma TDP-43 dosages have not yielded convincing results in differentiating FTLD-TDP from FTLD-Tau ^46– 51^.

If markers of the neuropathological process are far from being introduced in clinical practice, recent work has identified prognostic markers which will play an important role in forthcoming trials to assess treatment response. Above all, the assessment of neurofilament light chain (NfL), both in CSF and in blood, has recently gained great interest in the field of neurodegenerative disorders, with very high reliability of the assay obtained with advanced technologies ^52^. Even though increased NfL levels, reflecting axonal damage, seem to be non-specific for FTD, this marker appears to be a measure of disease intensity and predicts progression and survival ^44, 53– 57^. Longitudinal analysis of samples seems to suggest that levels change not long prior to symptom onset in genetic FTD, increasing by threefold to fourfold during conversion ^58^. Thus, a decrease in NfL levels could be a measure of successful disease modification in clinical trials ^59^.

Regarding *C9orf72* expansion carriers, the putative pathophysiological mechanisms include loss of *C9orf72* function as well as toxicity arising from the accumulation of sense and antisense transcripts of the expanded repeats. These RNA transcripts serve as templates for the synthesis of proteins of repeating dipeptides through repeat associated non-ATG (RAN) translation, as poly(GP) ^60– 62^. Increased CSF poly(GP) levels have been observed in *C9orf72* expansion carriers in both the presymptomatic and the symptomatic phase, suggesting that this marker might be altered long before the onset of clinical symptoms and could be useful as a preclinical biomarker in clinical trials in genetic FTD ^63– 65^.

Concomitantly to biomarker research, positron emission tomography (PET) techniques have paved the way for breakthroughs to assess pathophysiology *in vivo* in FTD ^66^. In particular, ** PET imaging of tau burden represents one of the most recent and promising applications in neurodegenerative dementias ^67^, and to date a number of tau tracers have been developed, such as [ ^18^F]flortaucipir (formerly known as [ ^18^F]AV1451), [ ^18^F]T807, and [ ^11^C]PBB3 ^68, 69^. However, studies of tau radioligands have so far not been proven to be particularly helpful in FTD, binding much more strongly to paired helical filament (PHF)-tau found mainly in Alzheimer’s disease ^70, 71^ than to other forms of tau found in the primary tauopathies such as progressive supranuclear palsy ^72– 75^ or corticobasal degeneration ^76– 78^. Only two *MAPT* mutations (V337M and R406W) are associated with PHF-tau and have shown strong binding with [ ^18^F]flortaucipir ^79^. New-generation tracers are currently under evaluation to overcome the non-specific/off-target binding, as binding has been reported in non-tau diseases such as in *C9orf72* mutation or svPPA, which are mainly associated with TDP-43 pathology ^80– 83^. Furthermore, the development of PET tracers able to specifically bind 3R or 4R tauopathies would have remarkable applications in clinical practice ^66^.

Considering that most tau tracers show non-specific/off-target binding with considerable overlap with TDP-43 pathology, the ongoing development of PET tracers specific for TDP-43 aggregates could have significant implications not only for FTD but also for ALS research ^84^. These ligands are expected to provide early and more accurate diagnosis of disease, help to monitor disease progression over time, and evaluate whether various therapeutic treatments are having a positive effect in individual patients.

## Looking for disease modulators

Postponing disease onset in FTD is a mandatory issue in the field, especially in presymptomatic individuals carrying pathogenetic mutations. Disease onset is highly variable in each of the genetic forms of FTD, with even intrafamilial variability ^85^.

A number of genetic modifiers have been identified in monogenic FTD, thanks to the development of collaborative international multicenter studies, such as GENFI in Europe and in Canada ( http://genfi.org.uk) ^86^ and ARTFL/LEFFTDS in the US ( https://ncrad.iu.edu/index.html) ^87^.

In *GRN* and *C9orf72* carriers, the *TMEM106B* genotype has been clearly associated with age at disease onset ^88– 90^. Another study of *C9orf72* carriers identified two overlapping genes ( *LOC101929163* and *C6orf10*) in which a polymorphism was associated with disease onset ^91^. The significance of the *C9orf72* repeat expansion length remains unclear, with no definitive evidence of an association with age of onset ^92– 97^. Other genes, such as small G-protein signaling modulator 3 ( *SGSM3*), which is involved in vesicular transport, have been identified as potential disease modifiers in *C9orf72* carriers ^98^. Little is known about factors that modify age at onset in *MAPT* mutation carriers ^99^.

Beyond genetic modifiers that are inherited and immutable, the possibility to intervene with environmental and other modulating factors is attractive. Some evidence shows that cognitive stimulating environments lead to brain volumetrical advantages and better cognitive performances in healthy individuals and in neurodegenerative disorders ^100^.

In the same view, in FTD patients, brain reserve, as measured by educational attainment, contributes to resilience against brain damage ^101– 103^. More interestingly, it has been recently demonstrated that highly educated at-risk subjects, carrying pathogenetic mutations associated with FTD, had better cognition and higher gray matter volume and showed slower loss of gray matter over time ^104, 105^. Thus, even in the presence of ongoing pathological processes, education may facilitate both brain reserve and brain maintenance in the presymptomatic phase of genetic FTD, virtually turning back the clock of the natural history of the disease. The demonstration that differences in early lifestyle may slow down later disease progression suggests that, even in monogenic disorders, outcomes are not completely determined from birth, and this opens up exciting perspectives for eventually delaying symptom onset ^104^.

## Disease-modifying therapeutic approaches

Clinical trial research in FTD spectrum disorders is in its infancy, and there are currently no approved disease-modifying therapies for sporadic or genetic FTD, but trials are now underway or planned for each of the three main FTD genetic mutations ^99, 106^.

*GRN* mutations, thought to act through loss-of-function mechanisms, are associated with decreased levels of the protein in serum and CSF ^107– 109^. Thus, restoring *GRN* function by targeting its receptors ^110, 111^ or increasing protein synthesis is a promising strategy ^112, 113^. In this view, an emerging target is sortilin, which serves as a lysosomal trafficking receptor for progranulin, and sortilin-mediated progranulin endocytosis has been implicated in FTD pathophysiology ^114^. Recombinant human anti-human sortilin monoclonal IgG1 antibodies (AL001) have been developed by Alector and are currently being investigated in a randomized controlled clinical trial (NCT03636204).

Antisense oligonucleotides (ASOs) are short oligonucleotide sequences that can disrupt RNA processing or transduction by activating RNA degradation or preventing ligation with particular RNA-binding proteins ^115^. One study in ALS patients found that the ASO drug ISIS 333611 was well tolerated ^116^, which suggests that ASOs could be beneficial in neurological disorder treatment ^117^.

Small molecule therapies and tau monoclonal antibodies are also being developed for tauopathies (with the potential for use in *MAPT* mutations) ^118^. Moreover, tau ASOs are also being planned for *MAPT* mutation carriers, with the objective of binding to tau messenger RNA and intercepting its translation, lowering the amount of tau protein in the brain. Currently, a clinical trial with a tau ASO (IONIS MAPTRx) is underway in mild Alzheimer’s disease (NCT03186989).

The development of new pharmacological interventions and precision-medicine approaches will help to steer clinical trials in FTD spectrum disorders in a productive direction ^119^.

## Conclusions

Several advances have been accomplished in recent years in the scenario of FTD. Both CSF and blood biomarkers as well as novel neuroimaging techniques are emerging as promising markers of disease pathophysiology and disease progression. Further effort is needed to identify preclinical biomarkers in the view of innovative clinical trials, in which disease-modifying therapies might need to be administered early in the disease course, as it is becoming clear also for Alzheimer’s disease. Indeed, up to now, only CSF and plasma progranulin levels for *GRN* carriers and CSF poly(GP) dipeptide repeat proteins for *C9orf72* expansion carriers seem to be significantly altered long before symptom onset ^99^, while neuroimaging abnormalities seem to become discernible about 10–15 years before symptom onset ^30, 120, 121^, as evaluated in large cohorts of patients which may lack single-subject applicability. Emerging neurophysiological techniques such as transcranial magnetic stimulation may be useful in the identification of disease progression, even in the presymptomatic phase of monogenic FTD, long before the onset of clinical symptoms ^107, 122– 124^.

Disease modulators, both genetic and environmental, which play an important role in disease onset and progression, may also become targets of therapeutic clinical trials, inducing modifications in the underlying pathophysiological cascade which results eventually in FTD. In this view, several innovative therapies such as ASOs, small molecules, and monoclonal antibodies are in the pipeline for the treatment of both sporadic and genetic FTD. Future research will certainly improve our knowledge of FTD and possibly open up a new era of disease-modifying therapies for this still-orphan disorder.

## Abbreviations

ALS, amyotrophic lateral sclerosis; ASO, antisense oligonucleotide; C9orf72, chromosome 9 open reading frame 72; CSF, cerebrospinal fluid; FTD, frontotemporal degeneration; FTLD, frontotemporal lobar degeneration; GRN, granulin; MAPT, microtubule-associated protein tau; NfL, neurofilament light chain; PET, positron emission tomography; PHF, paired helical filament; svPPA, semantic variant of primary progressive aphasia; TDP-43, TAR DNA-binding protein 43.
